# Molecular epidemiology of *Orientia tsutsugamushi* in chiggers and ticks from domestic rodents in Shandong, northern China

**DOI:** 10.1186/1756-3305-6-312

**Published:** 2013-10-29

**Authors:** Meng Zhang, Zhong-Tang Zhao, Hui-Li Yang, Ai-Hua Zhang, Xing-Qu Xu, Xiang-Peng Meng, Hai-Yu Zhang, Xian-Jun Wang, Zhong Li, Shu-Jun Ding, Li Yang, Lu-Yan Zhang

**Affiliations:** 1 Department of Epidemiology and Health Statistics, School of Public Health, Shandong University, Jinan, China; 2 Tai’an Center for Disease Control and Prevention, Tai’an, China; 3 Yinan Center for Disease Control and Prevention, Linyi, China; 4 Shandong Center for Disease Control and Prevention, Jinan, China

**Keywords:** Scrub typhus, *Orientia tsutsugamushi*, Chigger, Tick, Rodents

## Abstract

**Background:**

Scrub typhus is endemic to a 13,000,000-km^2^ area of the Asia-Pacific region, and causes an annual incidence of 1 million people. The mortality rate of scrub typhus ranges from 6.1% to 25% in Southeast Asia. Natural infection of *Orientia tsutsugamushi* has been identified in domestic rodents in Shandong Province. However, infestation of chiggers and ticks on the domestic rodents and prevalence and genotypes of *O. tsutsugamushi* in these Acarina remain unclear.

**Methods:**

During September 2010 to March 2012, 3134 chiggers and 89 ticks were collected from domestic rodents captured in three counties of Shandong Province. We amplified and sequenced the 56-kDa type-specific antigen gene of *O. tsutsugamushi* from DNA samples of these Acarina and designated to genotype according to sequence analysis.

**Results:**

Overall, the infestation rate of chiggers on domestic rodents was 17.0%, and the chigger index was 5.38. The infestation rate of ticks on rodents was 3.1%. Natural infection of *O. tsutsugamushi* was found in *Leptotrombidium taishanicum*, *L. linhuaikongense*, *L. intermedium*, *L. scutellare*, *L. palpale*, and *Ixodes* spp., the minimum positive rates of which were 5.9%, 3.2%, 1.2%, 0.8%, 0.8%, and 2.2%, respectively. Kawasaki-like genotypes were predominant in chiggers and ticks on domestic rodents, which were detected from *L. taishanicum*, *L. intermedium*, *L. scutellare*, *L. palpale*, and *Ixodes* spp. Shimokoshi-like genotype was detected from *L. palpale*.

**Conclusions:**

In the present study we investigated the infestation of chiggers and ticks on domestic rodents in Shandong Province, and identified the prevalence and genotypes of *O. tsutsugamushi* in the Acarina. Infestation of vector chiggers in domestic rodents, prevalence of *O. tsutsugamushi* in infested chiggers, and high nucleotide homologies among the *O. tsutsugamushi* sequences from the Acarina, their animal hosts and scrub typhus patients, implied that domestic rodents may play an important role in the transmission of scrub typhus in Shandong, China. Further studies are needed to verify the vector significance of chiggers and ticks that tested positive for *O. tsutsugamushi*, and to assess the risk of human exposure to chiggers and ticks on domestic rodents.

## Background

Scrub typhus is an acute febrile disease caused by *Orientia tsutsugamushi*, which is transmitted by larval-stage trombiculid mites. It is endemic to a 13,000,000-km^2^ area of the Asia-Pacific region, and causes an annual incidence of 1 million people [1,2]. The mortality rate of scrub typhus ranges from 6.1% to 25% in Southeast Asia [3,4]. Prevalence of scrub typhus and the burden of disease are usually underestimated. *O. tsutsugamushi* is a Gram-negative obligate intracellular bacterium categorized in the genus *Orientia* within the family *Rickettsiaceae*. In the recent decades, dramatic variation in phenotypes and genotypes of *O. tsutsugamushi* have been revealed in humans, animal hosts, and vector mites using immunological and molecular methods [5]. Highly polymorphic 56-kDa type-specific antigen (TSA), which consists of four variable domains (VD) I-IV presenting significant sequence heterogeneity among strains [6], is commonly used for type designation and evolutionary analysis of *O. tsutsugamushi*.

Scrub typhus has been endemic to southern China for decades, and it occurs mainly in summer and winter. The disease had not been reported in northern China until the first outbreak in Linyi City, Shandong Province in 1986 [7], and it occurs mainly in autumn and winter [8]; hitherto, sporadic cases and outbreaks have been reported nationwide [8]. According to the Shandong Diseases Reporting Information System, annual reported cases of scrub typhus have continuously increased in the past few years. In Shandong Province, the annual incidence of scrub typhus has increased from 0.23 in 2006 to 0.64 in 2012 per 100,000 people.

It was indicated that improper behavior at outdoor activities were responsible for scrub typhus [9,10]. Therefore, many researches focused on the prevalence of *O. tsutsugamushi* in chiggers parasitizing on wild rodents. The rate of positivity for *O. tsutsugamushi* ranged from 0.9% to 5.7% in chiggers collected from wild rodents [11-13]. A case-control study conducted in Shandong Province revealed that the living environment of poor sanitary conditions was significantly associated with the risk of scrub typhus [14]. In our previous study, we identified the prevalence of *O. tsutsugamushi* in domestic rodents, and proposed the hypothesis that domestic rodents may play an important role in the transmission of scrub typhus [15]. However, infestation of chiggers and ticks on domestic rodents and prevalence and genotype of *O. tsutsugamushi* in these Acarina remain unclear. To verify the hypothesis and provide a better understanding of the ecology and epidemiology of *O. tsutsugamushi*, chiggers and ticks collected from domestic rodents require screening for *O. tsutsugamushi*. In this study, the 56-kDa TSA gene of *O. tsutsugamushi* was amplified and sequenced from chiggers and ticks parasitizing on domestic rodents, to investigate the host range of *O. tsutsugamushi*, identify prevalent genotypes of *O. tsutsugamushi* in vectors, and evaluate the transmission risk of scrub typhus from a chigger or tick bite.

## Methods

### Study sites and ectoparasite collection

Shandong Province, located at the eastern coast of China, was a typical epidemic area of scrub typhus. It has a temperate and monsoonal climate. Xintai, Daiyue, and Yinan, characterized by mountains and hills, in middle Shandong were selected for investigation. Rodents were trapped from residences of farmers in rural areas every season from September 2010 to March 2012. Species of the captured mammals were identified and labeled. For each mammal, chiggers and ticks were removed from the ears, washed with TE buffer, and identified to species based on general features of external morphology.

### DNA preparation

We pooled 10-50 chiggers or 5-30 ticks of the same species collected from one animal host for homogenization with 0.6 ml ddH_2_O in a 1.5 ml Eppendorf tube. Ectoparasites of the same species from several animals of the same species at the same site and date were put together to make sure no less than 10 chiggers or 5 ticks were in a pool. The homogenates were added with 1/10 volume of lysis buffer (100 mM Tris-HCl, pH 8.0, 10 mM EDTA, 10%SDS) and 20 μL Proteinase K, and inoculated at 55°C for 1 hr. DNA was then extracted with equal volumes of phenol chloroform isoamylol (25:24:1) twice and precipitated by ethanol. After washing with 75% ethanol and drying, DNA was dissolved in 30 μL TE buffer (10 mM Tris-HCl, 1 mM EDTA, pH 8.0) and stored at -20°C for later usage.

### Nested polymerase chain reaction

Coding sequences covering VD I-III of 56-kDa TSA were amplified using nested PCR. Two sets of primers used were as follows: outer primers, comprising 34 (5’-TCAAGCTTATTGCTAGTGCAATGTCTGC-3’) and 55 (5’-AGGGATCCCTGCTGCTGTGCTTGCTGCG-3’) [16], and inner primers, comprising E (5’-GTTGGAGGAATGATTACTGG-3’) and B (5’-ACAGATGCACTATTAGGCAA-3’) [17].

The initial round of PCR was started with a 5-min denaturation at 94°C, followed by 30 cycles of 95°C for 30 sec, 57°C for 2 min, 70°C for 2 min, and then a final extension at 72°C for 10 min. The product was used as the template for the second round of PCR, which was started with a 3-min denaturation at 95°C, followed by 30 cycles of 95°C for 30 sec, 55°C for 30 sec, 72°C for 1 min, and then a final extension at 72°C for 10 min. The nested PCR products were visualized with an ultraviolet transilluminator after agarose gel electrophoresis and staining with ethidium bromide. Amplicons with a length of ≈ 733 bp were purified with Gel Extraction Kit (Omega, Norcross, GA, USA). DNA sequencing was carried out in an ABI 3730xl DNA Analyzer (Biosune, Shanghai, China).

### Phylogenetic analysis and sequence homologies

Complete or partial sequences encoding 56-kDa TSA of *O. tsutsugamushi* reference strains were retrieved from the GenBank (Table 1). Multiple sequence alignment was performed with Mega software using ClustalW algorithm. Phylogenetic relationships between *O. tsutsugamushi* strains from this study and the reference strains were inferred with Mega software by neighbor-joining method. Reliability of the phylogenetic analysis was evaluated using the bootstrap test on 1,000 replicates. Sequence homologies were calculated using MegAlign of Lasergene software (DNASTAR Inc., Madison, WI, USA).

**Table 1:** **Reference sequences of *Orientia tsutsugamushi *** **used in the phylogenetic analysis**

**Isolate**	**Source**	**Accession no.**	**Country**	**Year**	**Length (bp)**
Kato	Human	MM63382	Japan	1955	1590
Taiwan CDC Gilliam	-	DQ485289	Taiwan	-	1860
Karp	Human	M33004	New Guinea	1943	1599
Kuroki	Human	M63380	Japan	1981	1599
Kawasaki	Human	M63383	Japan	1981	1569
TA678	*Rattus rattus*	U19904	Thailand	1963	1548
TA763	*R. rattus*	U80636	Thailand	1963	1581
TA686	*Tupaia glis*	U80635	Thailand	1963	1599
TA716	*Menetes berdmorei*	U19905	Thailand	1963	1575
Shimokoshi	Human	M63381	Japan	1980	1565
Boryong	Human	L04956	South Korea	1998	1602
HSB1	Rodent	AF302983	Japan	1996-1997	1454
Yonchon	Human	U19903	South Korea	1989	1551
LX-1	*Leptotrombidium* spp.	AF173042	Japan	1986	1445
Lc-1	*L. chiangraiensis*	FJ374771	Thailand	2008	1590
LA-1	*L. arenicola*	AF173049	Malaysia	1993	1432
LF-1	*L. fletcheri*	AF173050	Malaysia	1993	1428
Fuji	*L. fuji*	AF201834	Japan	1998	1431
Sxh951	Human	AF050669	China	1998	1544
Neimeng-65	*Cricetulus barabansis*	DQ514319	China	2004-2005	1535
Shandong-XDM2	*L. scutellare*	DQ514320	China	1996	1591
CHL	Human	JX202567	China	2011	658
ZQL	Human	JX202576	China	2011	657
ZZF	Human	KC456647	China	2011	655
STAD10-24	*R. norvegicus*	JX202582	China	2010	656
STAD10-792	*Mus musculus*	JX202583	China	2010	689
TAXT11-1023	*M. musculus*	JX202584	China	2011	660
TAXT11-1103	*M. musculus*	JX202585	China	2011	706
TADY11-1168	*M. musculus*	JX202586	China	2011	676
TADY12-0305	*R. norvegicus*	JX202587	China	2012	661
TADY12-0307	*M. musculus*	JX202588	China	2012	660
YN11-24	*C. barabansis*	KC456651	China	2011	667

### Nucleotide sequence accession numbers

DNA sequences of *O. tsutsugamushi* obtained in this study were deposited into GenBank under the accession no. KC456648-KC456650, KC456657- KC456660, and KF421129 (Table 2).

**Table 2:** **Sequences encoding partial 56-kDa type-specific antigen of *Orientia tsutsugamushi *** **determined in this study**

**Sample code**	**Source**	**Accession no.**	**Location**	**Sampling date**	**Length (bp)**
YNM1	*L. palpale*	KC456648	Yinan	Mar-2011	662
YNM2	*L. intermedium*	KC456649	Yinan	Jan-2011	666
YNM5	*L. taishanicum*	KC456650	Yinan	Jan-2011	669
TAM1	*L. palpale*	KC456657	Tai’an	Oct-2010	657
TAM2	*L. palpale*	KC456658	Tai’an	Oct-2010	669
TAM3	*L. palpale*	KC456659	Tai’an	Oct-2011	662
TAM5	*L. scutellare*	KC456660	Tai’an	Oct-2011	656
TAT1	*Ixodes* spp.	KF421129	Tai’an	Oct-2011	657

### Ethical statement

The study was approved by the Ethics Committee on Preventive Medicine of Shandong University.

## Results

### Infestation of Acarina on domestic rodents

**Figure 1:**
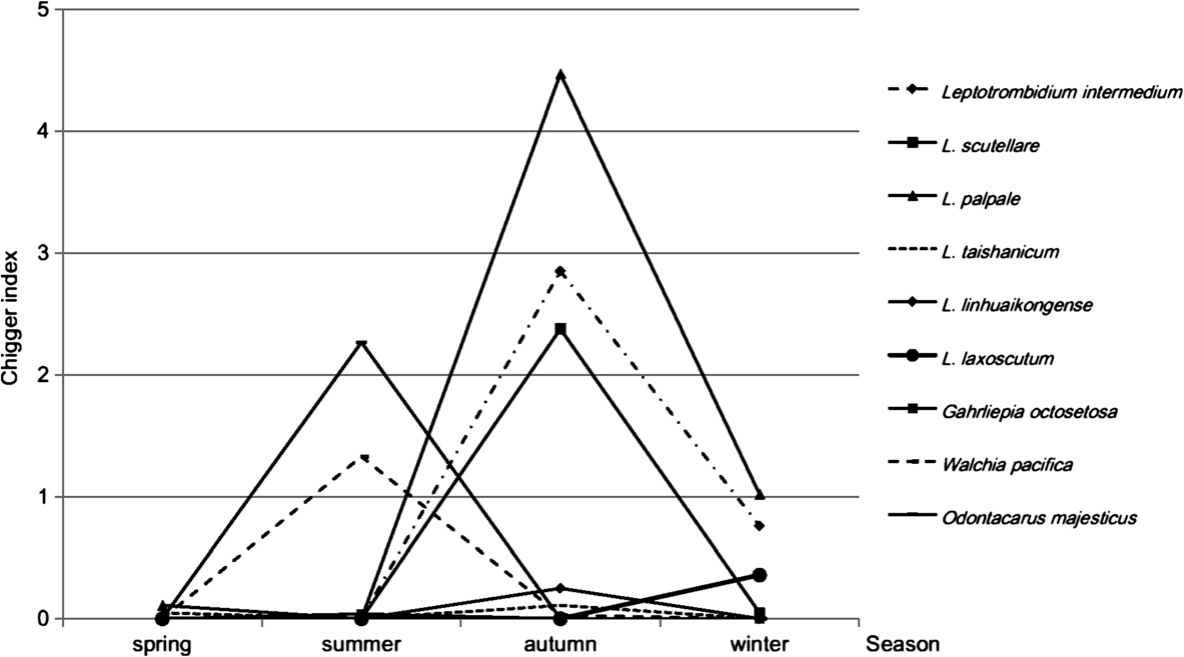
Seasonal fluctuation of chigger mites on domestic rodents in Shandong, northern China.

During the study period, 583 domestic rodents were captured, including 359 *Rattus norvegicus*, 222 *Mus musculus*, and 2 *Rattus rattus*. We collected 3134 chigger mites from the ears of the captured mammals, including *L. taishanicum* (34, 1.1%), *L. linhuaikongense* (62, 2.0%), *L. intermedium* (817, 26.1%), *L. scutellare* (608, 19.4%), *L. palpale* (1275, 40.7%), *L. laxoscutum* (46, 1.5%), *Walchia pacifica* (112, 3.6%), *Odontacarus majesticus* (177, 5.6%), and *Gahrliepia octosetosa* (3, 0.1%). Rates of chigger infestation on domestic rodents in spring, summer, autumn and winter were 4.7%, 15.4%, 27.0%, and 10.4%, respectively (Table 3). Seasonal fluctuation of chigger mites during the four seasons was shown in Figure 1. *L. palpale*, *L. intermedium*, and *L. scutellare* were predominant chigger species in autumn and winter, while *O. majesticus* and *W. pacifica* were predominant in summer. Overall, the infestation rate of chigger mites on the captured domestic rodents was 17.0%, and the chigger index was 5.38. Among the 359 *R. norvegicus*, 72 (20.1%) were found to be parasitized by chiggers, with a chigger index of 8.73. The infestation rate of chiggers in *M. musculus* was 12.2%, and the chigger index was 2.74. Chigger densities of different species collected from different hosts were demonstrated in Table 4. *L. intermedium*, *L. scutellare*, and *L. palpale* were the first three predominant species of chiggers from both *R. norvegicus* and *M. musculus*. No chigger was examined from *R. rattus*. In addition, 89 ticks of *Ixodes* spp. were collected from 18 rodents. The infestation rate of ticks on domestic rodents was 3.1%.

**Table 3:** Rates of chigger infestation on domestic rodents, northern China

**Mammal species**	**Season, no. infested/no. captured (%)**				
	**Spring**	**Summer**	**Autumn**	**Winter**	**Total**
*Rattus norvegicus*	4/99 (4.0)	12/52 (23.1)	49/151 (32.5)	7/57 (12.3)	72/359 (20.1)
*Mus musculus*	2/29 (6.9)	0/26 (0)	19/101 (18.8)	6/66 (9.1)	27/222 (12.2)
*R. rattus*	0	0	0	0/2 (0)	0/2 (0)
Total	6/128 (4.7)	12/78 (15.4)	68/252 (27.0)	13/125 (10.4)	99/583 (17.0)

**Table 4:** Species of chiggers collected from domestic rodents, northern China

**Chigger species**	**Mammal species, no. chiggers (chigger index)**			
	***Rattus norvegicus***	***Mus musculus***	***R. rattus***	**Total**
	**(n = 359)**	**(n = 222)**	**(n = 2)**	**(n = 586)**
*Leptotrombidium intermedium*	601 (1.67)	216 (0.97)	0	817 (1.39)
*L. scutellare*	426 (1.19)	182 (0.82)	0	608 (1.04)
*L. palpale*	1078 (3.00)	197 (0.89)	0	1275 (2.18)
*L. taishanicum*	30 (0.08)	4 (0.02)	0	34 (0.06)
*L. linhuaikongense*	62 (0.17)	0	0	62 (0.11)
*L. laxoscutum*	42 (0.12)	4 (0.02)	0	46 (0.08)
*Gahrliepia octosetosa*	3 (0.01)	0	0	3 (0.01)
*Walchia pacifica*	106 (0.30)	6 (0.03)	0	112 (0.19)
*Odontacarus majesticus*	177 (0.49)	0	0	177 (0.30)
Total	2525 (7.03)	609 (2.74)	0	3134 (5.35)

### Prevalence of *O. tsutsugamushi* in chiggers and ticks

One hundred and sixty four pools of mites were screened for *O. tsutsugamushi* using nested PCR. Among the 29 positive mite homogenates determined by nested PCR, 24 pools were collected from *R. norvegicus*, and 5 from *M. musculus*. Natural infection of *O. tsutsugamushi* was found in *L. taishanicum*, *L. linhuaikongense*, *L. intermedium*, *L. scutellare*, and *L. palpale*, the minimum positive rates (MPR = positive pools/total chigger counts) of which were 5.9%, 3.2%, 1.2%, 0.8%, and 0.8%, respectively. The MPRs of chigger mites in spring, summer, autumn, and winter were 10%, 0, 0.9%, and 1.8%, respectively (Table 5). The collected ticks were divided into 12 pools, of which 2 pools were positive for *O. tsutsugamushi*.

**Table 5:** **Minimum positive rate of *Orientia tsutsugamushi *** **in chigger mites collected from domestic rodents, northern China**

**Chigger species**	**Season, no. positive pools/total counts (%)**				
	**Spring**	**Summer**	**Autumn**	**Winter**	**Total**
*Leptotrombidium intermedium*	0	0	9/721 (1.2)	1/96 (1.0)	10/817 (1.2)
*L. scutellare*	0	0	4/602 (0.7)	1/6 (16.7)	5/608 (0.8)
*L. palpale*	1/14 (7.1)	0	6/1132 (0.5)	3/129 (2.3)	10/1275 (0.8)
*L. taishanicum*	1/6 (16.7)	0	1/28 (3.6)	0	2/34 (5.9)
*L. linhuaikongense*	0	0	2/62 (3.2)	0	2/62 (3.2)
*L. laxoscutum*	0	0	0	0/46 (0)	0/46 (0)
*Gahrliepia octosetosa*	0	0/3 (0)	0	0	0/3 (0)
*Walchia pacifica*	0	0/104 (0)	0/8 (0)	0	0/112 (0)
*Odontacarus majesticus*	0	0/177 (0)	0	0	0/177 (0)
Total	2/20 (10.0)	0/284 (0)	22/2553 (0.9)	5/277 (1.8)	29/3134 (0.9)

### Phylogenetic analysis and sequence homologies

**Figure 2:**
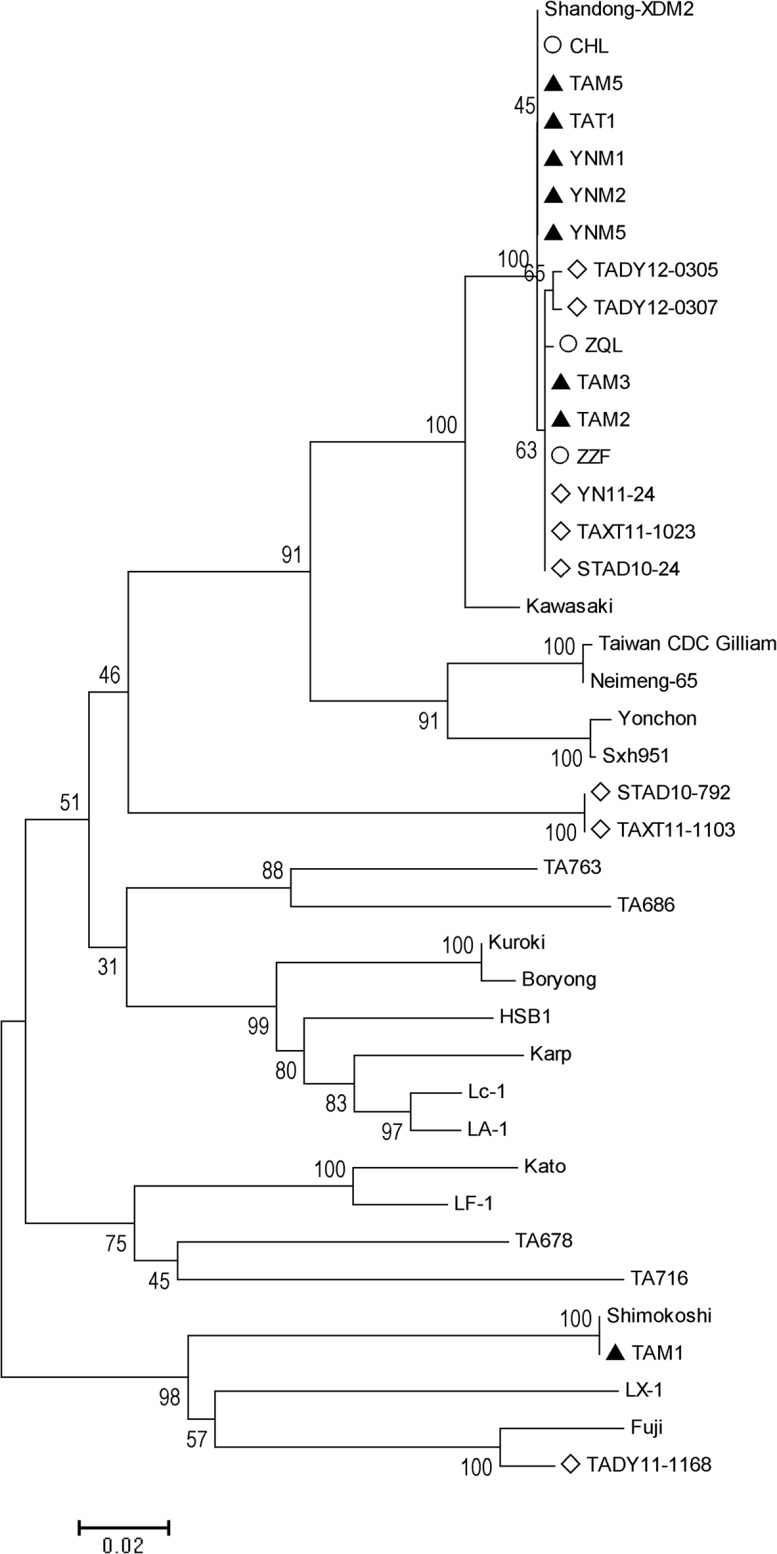
**Phylogenetic tree based on partial 56-kDa type-specific antigen gene of *Orientia tsutsugamushi*.** The phylogenetic analysis was performed using the neighbor-joining method with Kimura 2-parameter distance model. Solid triangle indicates sequences determined in this study. Hollow circle and diamond indicate sequences previously determined in scrub typhus patients and domestic rodents in the study area. Scale bar indicates genetic distance.

Seven sequences of *O. tsutsugamushi* from chiggers and 1 from ticks determined in this study, 11 sequences previously determined in patients and domestic rodents in the same study area, and 21 reference sequences of *O. tsutsugamushi* were enrolled in the phylogenetic analysis. The *O. tsutsugamushi* harbored by chigger mites were related to either Kawasaki or Shimokoshi strain originating from Japan (Figure 2). TAM1 had 99.4% nucleotide identity to Shimokoshi strain, and denominated as SKS genotype. TAM5, YNM1, YNM2, YNM5, and TAT1 were identical to CHL (KWS1 genotype), which had 96.5% nucleotide identity to Kawasaki strain. TAM2 and TAM3 were identical to STAD10-24 (KWS2 genotype), which had an identity of 96.4% to Kawasaki strain. The nucleotide sequence identities ranged from 70.7% to 100% among the 8 sequences. The *O. tsutsugamushi* sequences determined from chiggers and ticks had identities ranging from 70.5% to 100% with those previously identified in humans, and 69.6% to 100% with those in domestic rodents in the study area (Table 6). TAM2, which was identified from the chiggers parasitizing on *R. norvegicus* STAD10-24, was identical to *O. tsutsugamushi* sequence carried by the same rodent. TAM5, which was identified in the mite pools collected from *M. musculus* TAXT11-1023, showed 99.9% identity with *O. tsutsugamushi* sequence carried by the same rodent. TAM3 detected in chiggers and TAT1 in ticks, which were collected from the same rodent, had an identity of 99.9% with each other. The nucleotide homologies of partial 56-kDa TSA gene between *O. tsutsugamushi* determined in this study and reference strains were shown in Table 6.

**Table 6:** **Nucleotide homologies of partial 56-kDa TSA gene between *Orientia tsutsugamushi *** **strains**

**Reference strain**	**Homologies (%)**							
	**TAM1**	**TAM2**	**TAM3**	**TAM5**	**YNM1**	**YNM2**	**YNM5**	**TAT1**
Kawasaki	69.8	96.4	96.4	96.5	96.5	96.5	96.5	96.5
Shimokoshi	99.4	70.4	70.4	70.4	70.4	70.4	70.4	70.4
Shandong-XDM2	71.2	99.9	99.9	100	100	100	100	100
CHL	70.7	99.9	99.9	100	100	100	100	100
ZQL	70.5	99.9	99.9	99.7	99.7	99.7	99.7	99.7
ZZF	70.7	100	100	99.9	99.9	99.9	99.9	99.9
TADY12-0305	70.3	99.6	99.6	99.4	99.4	99.4	99.4	99.4
TADY12-0307	70.4	99.6	99.6	99.4	99.4	99.4	99.4	99.4
YN11-24	70.5	99.9	99.9	99.7	99.7	99.7	99.7	99.7
TAXT11-1023	70.7	100	100	99.9	99.9	99.9	99.9	99.9
STAD10-24	70.7	100	100	99.9	99.9	99.9	99.9	99.9
STAD10-792	69.6	76.4	76.4	76.4	76.4	76.4	76.4	76.4
TAXT11-1103	69.6	76.4	76.4	76.4	76.4	76.4	76.4	76.4
TADY11-1168	75.7	70.0	70.0	70.0	70.0	70.0	70.0	70.0

## Discussion

Vectors for *O. tsutsugamushi* require four prerequisites: 1) epidemiological correlation with the occurrence of scrub typhus; 2) natural infection of *O. tsutsugamushi*; 3) could feed on the hosts and transmit the agent; and 4) transovarial transmission of the agent. Six species of chigger mites has been confirmed as the vectors for *O. tsutsugamushi* in China, which were *Leptotrombidium deliense*, *L. insularae*, *L. kaohuensis*, *L. rubellum*, *L. jishoum*, and *L. scutellare*[18]. *O. tsutsugamushi* DNA was detected in *L. taishanicum*, *L. linhuaikongense*, *L. intermedium*, *L. scutellare*, *L. palpale* and *Ixodes* spp. in this study. To our knowledge, it is the first study to identify *O. tsutsugamushi* in *L. taishanicum* and *Ixodes* spp.. It was reported that *O. tsutsugamushi* had been successfully isolated from *Ixodes granulatus* collected from domestic rodents; however, artificial infection experiments showed that the agent could live for no more than 4 days in the ticks [18]. It was possible that some species of *O. tsutsugamushi*-positive chiggers and ticks acquired the agent from infected hosts, but were not able to support their survival or proliferation.

We demonstrated that three genotypes of *O. tsutsugamushi* were circulating in the chiggers and ticks collected from domestic rodents in Shandong Province, which were KWS1, KWS2, and SKS. KWS1 genotype was detected from *L. taishanicum*, *L. intermedium*, *L. scutellare*, *L. palpale*, and *Ixodes* spp.. KWS2 genotype was identified in *L. scutellare* and *L. palpale*. And SKS genotype was found in *L. palpale*. *O. tsutsugamushi* DNA was detected from two pools of *L. linhuaikongense*; however, we failed to identify the genotypes because of unsuccessful sequencing.

The majority of scrub typhus cases occurred in October and November in Shandong Province [8]. *L. palpale*, *L. intermedium*, and *L. scutellare* were predominant mite species parasitizing on domestic rodents during autumn in Shandong. *L. scutellare* has been confirmed to be a vector for scrub typhus. KWS1 genotype of *O. tsutsugamushi* was detected from *L. intermedium*; however, the low infestation rate to humans makes it of less significance as a vector for scrub typhus*.* Genotypes of *O. tsutsugamushi* are usually restricted to a specific chigger species [19]. *L. palpale* could be infested with KWS1, KWS2, and SKS genotypes of *O. tsutsugamushi*, and may be a possible vector of public health importance. *Odontacarus majesticus* and *Walchia pacifica* were predominant during the summer. It was reported that *Odontacarus majesticus* and *Walchia pacifica* could be infested with *O. tsutsugamushi* in China [20,21]. Scrub typhus cases were sporadically reported in summer in Shandong Province. Although no *O. tsutsugamushi* DNA was detected from the two species of chiggers in this study, their potential to be vectors for in-house transmission of scrub typhus should not be excluded. The vector competence of the above mentioned *O. tsutsugamushi*-positive chiggers and ticks requires further evaluation.

Shimokoshi-type *O. tsutsugamushi* presented weak virulence to mice [22]; however, the resulting clinical manifestations in humans need more documentation. Scrub typhus cases of Shimokoshi type were first reported in Niigata Prefecture, Japan in 1984 [22], and recently reported in northeastern and western Japan [23]. Hitherto, scrub typhus cases caused by Shimokoshi-like genotypes of *O. tsutsugamushi* have not been reported in China. Detection of Shimokoshi-like genotype of *O. tsutsugamushi* in *L. palpale* is an alert to potential scrub typhus outbreaks of this genotype in humans. It was reported that Shimokoshi type had low cross reactivity with the prototype strains of Kato, Karp, and Gilliam [22]. Currently used diagnostic reagents of scrub typhus in China, with the three prototype strains as antigens, is not adequate to detect Shimokoshi-type *O. tsutsugamushi*. In order to identify the clinical profile of scrub typhus cases caused by Shimokoshi-like genotypes of *O. tsutsugamushi*, Shimokoshi strain should be added in the antigen pool of diagnostic tests.

Rodents were found to be active in the living room, kitchen, toilet, and yard in the study area. Natural infection of *O. tsutsugamushi* was identified in domestic rodents captured in Shandong, China [15]. *O. tsutsugamushi* DNA was detected in Acarina parasitizing on domestic rodents, and had high identities with those detected in the domestic rodents and in scrub typhus patients. It was indicated that chiggers had the opportunity to get infected from the domestic rodents, be taken to wherever the rodents go, and transmit the agent to their offspring. The residents could be infected with scrub typhus through occasional bites of the infected chiggers. Conclusions of the present study supported our previous hypothesis that domestic rodents play an important role in the transmission of scrub typhus.

However, the results should be interpreted under some limitations. First, chiggers usually parasitize on the whole body surface of animal hosts, especially on ears, breasts, genitals, and anus; however, only those that were parasitizing on the ears were collected in this study. The actual infestation of chiggers on domestic rodents and chigger index were likely to be larger than those indicated in the study. Second, considering the copies of *O. tsutsugamushi* from individual chigger were too low for PCR detection, we screened for *O. tsutsugamushi* from chigger pools rather than individual mite. The actual rate of positivity for *O. tsutsugamushi* in chiggers was no less than that indicated by MPR. Besides, there is a possibility that more than one genotype strain of *O. tsutsugamushi* existing in a pool, but only the predominant type could be detected in the study. Third, we failed to identify the collected ticks into species, which is required in future research.

## Conclusions

In the present study, we identified the prevalence of *O. tsutsugamushi* in chiggers and ticks collected from domestic rodents in Shandong, China. High nucleotide homologies were found among the *O. tsutsugamushi* sequences from the collected Acarina, their animal hosts and scrub typhus patients. The results indicated a possibility that chiggers and ticks acquire *O. tsutsugamushi* from the domestic rodents, and transmit it to humans, which supported our hypothesis that domestic rodents may play an important role in the transmission of scrub typhus in Shandong, China. Deracination inside the residences would be a necessary and effective measure to reduce the transmission risk of scrub typhus. Further studies are needed to verify the vector significance of chiggers and ticks that tested positive for *O. tsutsugamushi*, and to assess the risk of human exposure to chiggers and ticks on domestic rodents.

## Abbreviations

TSA: Type-specific antigen; VD: Variable domain; O. tsutsugamushi: *Orientia tsutsugamushi*; R. rattus: *Rattus rattus*; M. musculus: *Mus musculus*; R. norvegicus: *Rattus norvegicus*; L. taishanicum: *Leptotrombidium taishanicum*; L. intermedium: *Leptotrombidium intermedium*; L. scutellare: *Leptotrombidium scutellare*; L. palpale: *Leptotrombidium palpale*; L. linhuaikongense: *Leptotrombidium linhuaikongense*; L. laxoscutum: *Leptotrombidium laxoscutum*; W. pacifica: *Walchia pacifica*; O. majesticus: *Odontacarus majesticus*; and G. octosetosa: *Gahrliepia octosetosa*

## Competing interests

The authors declare that they have no competing interests.

## Authors' contributions

MZ, ZTZ, and XJW were responsible for the conception and design of the study. MZ, ZL, HLY, AHZ, XPM, XQX, HYZ, and SJD carried out rodent capture, ectoparasite collection, and morphological identifications of chiggers and ticks. MZ, LY, and LYZ carried out detection of *O. tsutsugamushi* and bioinformatics process. All the authors participated in manuscript drafting, and approved the final manuscript.
